# Caveolin‐1 inhibition mediates the opposing effects of alcohol on γ‐secretase activity in arterial endothelial and smooth muscle cells

**DOI:** 10.14814/phy2.15544

**Published:** 2023-01-12

**Authors:** Naresh K. Rajendran, Weimin Liu, Paul A. Cahill, Eileen M. Redmond

**Affiliations:** ^1^ Department of Surgery University of Rochester Medical Center Rochester New York USA; ^2^ Vascular Biology and Therapeutics Laboratory, School of Biotechnology Dublin City University Dublin Ireland

## Abstract

Notch is important to vessel homeostasis. We investigated the mechanistic role of caveolin‐1 (Cav‐1) in mediating the effects of alcohol (Ethanol/EtOH) on the γ‐secretase proteolytic activity necessary for Notch signaling in vascular cells. Human coronary artery endothelial cells (HCAEC) were treated with EtOH (0–50 mM), Notch ligand delta‐like ligand 4 (Dll4), and the γ‐secretase inhibitor DAPT. EtOH stimulated Notch signaling in HCAEC as evidenced by increased Notch receptor (N1, N4) and target gene (hrt2, hrt3) mRNA levels with the most robust response achieved at 25 mM EtOH. Ethanol (25 mM) stimulated γ‐secretase proteolytic activity, to the same extent as Dll4, in HCAEC membranes. Ethanol inhibited Cav‐1 mRNA and protein levels in HCAEC. Caveolin‐1 negatively regulated γ‐secretase activity in HCAEC as Cav‐1 knockdown stimulated it, while Cav‐1 overexpression inhibited it. Moreover, Cav‐1 overexpression blocked the stimulatory effect of EtOH on γ‐secretase activity in HCAEC. Although EtOH also inhibited Cav‐1 expression in human coronary artery smooth muscle cells (HCASMC), EtOH inhibited γ‐secretase activity in HCASMC in contrast to its effect in HCAEC. The inhibitory effect of EtOH on γ‐secretase in HCASMC was mimicked by Cav‐1 knockdown and prevented by Cav‐1 overexpression, suggesting that in these cells Cav‐1 positively regulates γ‐secretase activity. In conclusion, EtOH differentially regulates γ‐secretase activity in arterial EC and SMC, being stimulatory and inhibitory, respectively. These effects are both mediated by caveolin‐1 inhibition which itself has opposite effects on γ‐secretase in the two cell types. This mechanism may underlie, in part, the effects of moderate drinking on atherosclerosis.

## Graphical abstract

Alcohol (EtOH) inhibits caveolin‐1 (Cav‐1) expression in both endothelial cells (EC) and smooth muscle cells (SMC). Caveolin‐1 negatively regulates gamma‐secretase activity in EC, whereas it positively regulates gamma‐secretase activity in SMC. Thus, alcohol‘s inhibition of Cav‐1 expression in EC and SMC results in increased and decreased Notch signaling, respectively. These differential changes in Notch signaling in EC and SMC would be expected to act synergistically to maintain vessel homeostasis and prevent arteriosclerosis.

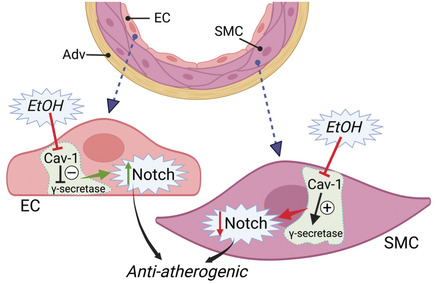

**NEW AND NOTEWORTHY**

These data highlight a differential regulation by alcohol of γ‐secretase activity in arterial endothelial versus smooth muscle cells, and uncover the common mediating mechanism involved, that is, caveolin‐1 inhibition. Given the important role of γ‐secretase/Notch signaling in arterial homeostasis, these effects are likely pertinent to cardiovascular health in alcohol consumers.

## 1 INTRODUCTION

Epidemiologic studies indicate a complex relationship between alcohol (EtOH) consumption and disease, especially arteriosclerotic cardiovascular disease. When compared to teetotalers, alcohol abuse and heavy binge drinking (giving rise to blood alcohol concentration/BAC >35 mM) are associated with exacerbated arterial disease and related mortality and morbidity due to heart attacks and strokes. In contrast, frequent low‐to‐moderate alcohol consumption, generally considered in the range 1–3 drinks/day (BAC 5–25 mM) seems protective (Colpani et al., 2018; Costanzo et al., 2010; Fernandez‐Sola, 2015; McEvoy et al., 2022). Studies in mouse models are in general agreement with these human epidemiologic data (Furuta et al., 2019; Gil‐Bernabe et al., 2011; Liu et al., 2011). However, the precise cell and molecular mechanisms involved in mediating alcohol's effects are not fully understood.

**FIGURE 1:**
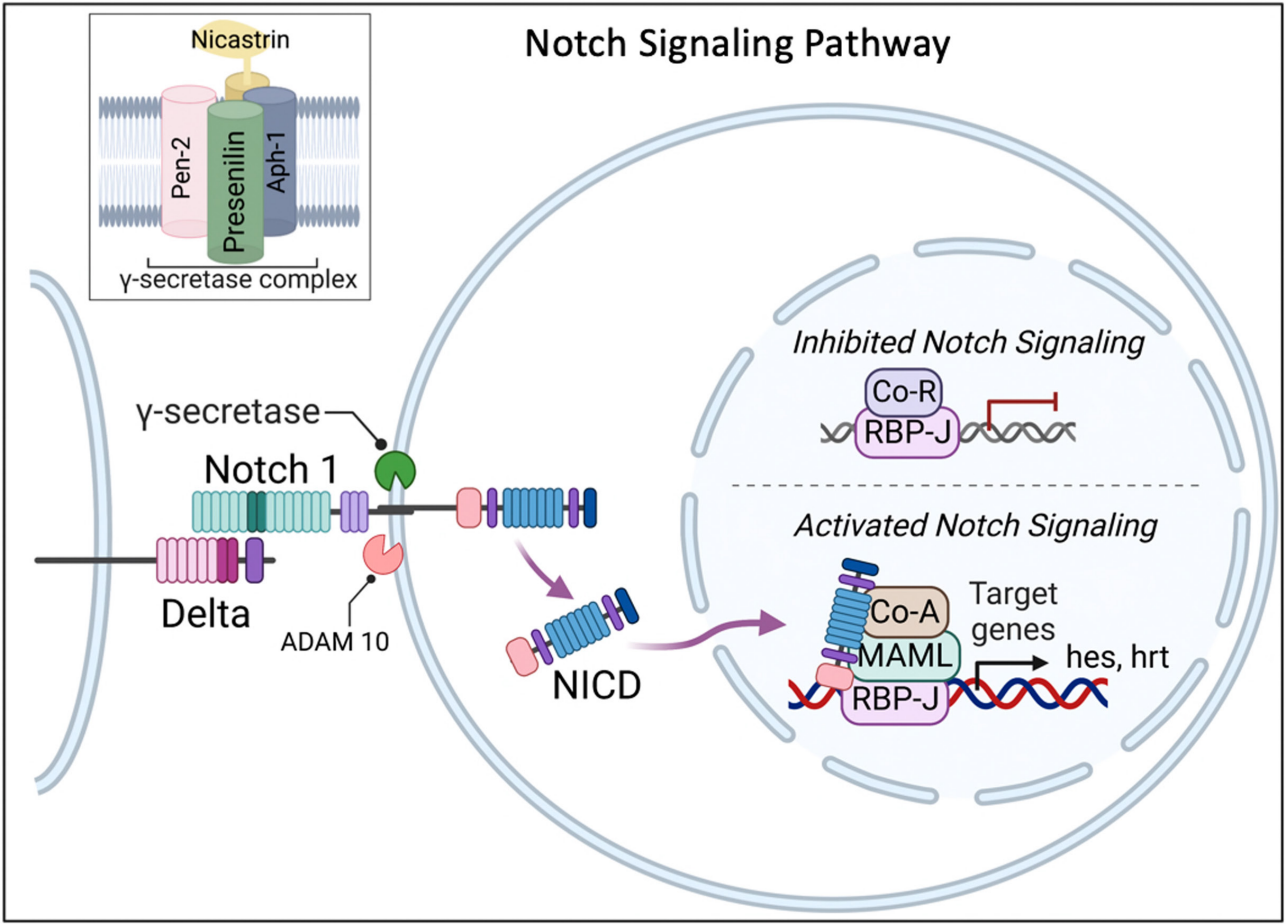
Notch signaling pathway, and γ‐secretase complex (inset box). Figure created using Biorender.

Notch is critical in the development and homeostasis of multiple tissues, and alteration of its signaling is thought to play a role in cardiovascular disease, immune disorders, and cancers (Siebel & Lendahl, 2017; Zhou et al., 2022). With respect to cardiovascular disease, vascular endothelial Notch signaling is mechanosensitive and deters arteriosclerosis by positively influencing endothelial cell (EC) quiescence, junctional stability, and release of bioactive compounds (Mack & Iruela‐Arispe, 2018; Rajendran et al., 2021). On the other hand, Notch signaling in vascular smooth muscle cells affects their phenotype and proliferation in a manner considered pro‐atherogenic (Morrow et al., 2003, 2005). Together, these Notch‐dependent endothelial and smooth muscle effects are influential in vessel homeostasis and in modulating arteriosclerotic lesion development (Davis‐Knowlton et al., 2019; Li et al., 2009; Souilhol et al., 2020). In the classic pathway, interaction of the Notch receptor (i.e., N1–4) with a ligand (i.e., Delta‐like 1, −3, −4, or Jagged1, 2) initiates sequential proteolytic cleavage of the receptor by α‐secretase (ADAM 10), followed by γ‐secretase, resulting in the release of Notch intracellular domain (NICD) from the cytoplasmic side of the cell membrane (Artavanis‐Tsakonas et al., 1999) (Figure 1). NICD can then be routed into the nucleus where it binds the transcription factor RBP‐J and forms a ternary complex with co‐activators including mastermind‐like‐1 (MAML‐1) resulting in transcriptional activation of Notch target genes such as Hes and Hrt (Artavanis‐Tsakonas et al., 1999; Figure 1). The intramembrane protease γ‐secretase is a hetero‐trimeric protein complex with presenilin (PSN‐1, PSN‐2) as the catalytic subunit, and accessory subunits pre‐senilin enhancer‐2 (Pen‐2), anterior pharynx defective‐1 (Aph‐1), and Nicastrin (Shih Ie & Wang, 2007; Figure 1, insert box). In addition to Notch, numerous additional substrates of γ‐secretase have been identified in the last 20 years, bolstering its potential biological importance.

We have previously reported differential effects of EtOH (at levels associated with moderate drinking, i.e., 15–25 mM) on Notch signaling in vascular cells (Morrow et al., 2008, 2010). Specifically, EtOH inhibits Notch signaling in smooth muscle cells (SMC) (Morrow et al., 2010) in part by inhibiting γ‐secretase activity in the absence of any effect on α‐secretase activity (Hatch et al., 2015). In direct contrast to its effect in SMC, EtOH promotes Notch signaling in EC (Morrow et al., 2008, 2014). Whether it does this by stimulating γ‐secretase activity in endothelium is not known and is the focus of this study.

Of interest, caveolin‐1, the scaffolding protein of caveolae involved in the regulation of numerous signaling pathways (Frank et al., 2003), has been reported to both positively and negatively regulate Notch signaling and γ‐secretase activity in neural and mesenchymal stem cells (Kapoor et al., 2010; Li et al., 2011; Wang et al., 2013). Caveolin‐1's effect on γ‐secretase in arterial cells has not been established. We report here for the first time that, in contrast to its effects in SMC, EtOH stimulates γ‐secretase activity in EC, and moreover, that the differential effects of EtOH on γ‐secretase in vascular EC and SMC are, in both cases, mediated by inhibition of caveolin‐1 expression in these cells.

## 2 MATERIALS AND METHODS

### 2.1 Experimental design

Human arterial cells, in which caveolin‐1 expression was experimentally manipulated, were exposed to ethanol before γ‐secretase activity was assessed by fluorogenic peptide assay.

### 2.2 Cell culture

Human coronary artery endothelial cells (HCAEC) and human coronary artery smooth muscle cells (HCASMC) were obtained from Lonza Inc, Allendale, NJ. HACEC were cultured in optimized EGM‐2 medium (cat# CC‐3162, Clonetics**®**, Lonza) and HCASMC were grown in optimized SmGM‐2 media (cat# CC‐3182, Clonetics**®**, Lonza). HCAEC passages 5–14 and HCASMC passages 4–10 were used in experiments and culture medium was renewed every 2 days. Cells were grown on fibronectin‐coated 10‐cm dishes covered in parafilm and maintained in a standard humidified incubator at 37°C in a 5% CO_2_ atmosphere. Cells were treated with/without experimental agents as indicated (Ethanol 5–50 mM; DLL4, 3 μg/ml; DAPT, 20 μM). Under these conditions, there was little change (<10%) in ethanol concentration (determined by diagnostic kit from Sigma‐Aldrich) after 24‐h incubation +/− HCAEC or HCASMC indicating no significant metabolism of ethanol by these cells.

### 2.3 Quantitative real‐time reverse transcription polymerase chain reaction

Total RNA was extracted from the cells using RNeasy Mini Kit (cat# 74134) and 1 μg RNA used for cDNA synthesis (iScript cDNA Synthesis kit; cat# 1708891, Bio‐Rad). SYBR Green master mix (cat# 4309155, ThermoFisher Scientific) was used to determine mRNA expression according to the manufacturer's protocol using a QuantStudio 3 (Applied Biosystems). The RT‐PCR cycling conditions were as follows: DNA polymerase activation at 95°C for 10 min, followed by 40 PCR cycles, each cycle consisting of 95°C for 15 s (denature) and 60°C for 1 min (anneal/extend). GAPDH was used as a housekeeping control and data were analyzed with Applied Biosystems® qPCR analysis software (ThermoFisher Scientific).

### 2.4 Primer sequences

**Table:** 

Gene	Forward primer	Reverse primer
Notch1	5′‐CAGGGTGTGCACTGTGAGAT‐3′	5′‐GACAGGCACTCGTTGACATC‐3′
Notch4	5′‐CTAGGGGCTCTTCTCGTCCT‐3′	5′‐CAACTTCTGCCTTTGGCTTC‐3′
Hrt2	5′‐GTACCTGAGCTCCGTGGAAG‐3′	5′‐AGTTGTGGAGAGGCGACAAG‐3′
Hrt3	5′‐GGTGGGACAGGATTCTTTGA‐3′	5′‐AGCTGTTGAGGTGGGAGAGA‐3′
Caveolin‐1	5′‐GCTGTCGGAGCGGTTAGTT‐3′	5′‐TGTAGATGTTGCCCTGTTCCC‐3′
Aph1	5′‐GTCGCAGGGAAGGCAGATGA‐3′	5′‐GGGATCTGTCAGGCGATCAG‐3′
Aph2	5′‐ATGTCTTCACCATCGCCACC‐3′	5′‐CATAGGCCAGCAGTCGCATA‐3′
Nicastrin	5′‐ACTAGCAGGTTTGTGCAGGG‐3′	5′‐GTAGACCATCCTCGAGCTGC‐3′
Presenilin‐1	5′‐GCGGGGAAGCGTATACCTAA‐3′	5′‐ACGTACAGTATTGCTCAGGTG‐3′
Presenilin‐2	5′‐CAAATACGGAGCGAAGCACG‐3′	5′‐AAGGCTCCCCAAAACTGTCA‐3′
PEN2	5′‐GAAGTGAGCTCTCCTGGGTCA‐3′	5′‐TCATTGGACACTCGCTCCAG‐3′
GAPDH	5′‐CGAGATCCCTCCAAAATCAA‐3′	5′‐TTCACACCCATGGACGAACAT‐3′

### 2.5 γ‐Secretase assay

γ‐secretase activity in vascular cells was determined using a fluorogenic peptide substrate (C‐terminal fragment of β‐APP) as described previously (Hatch et al., 2018). The ReadyPrep (membrane II) protein extraction kit (cat# 163–2084 BioRad) was used to isolate cellular membranes (from HCAEC and HCASMC) and then to extract associated integral membrane proteins into a solution. Protein concentration in membrane preparations was determined by Bradford assay. Activity was measured by pre‐incubating solubilized membranes (40 μg of total protein in assay buffer with 0.25% CHAPSO w/v), +/− DLL4 (R&D Systems, Minneapolis, MN), +/− ethanol (25 mM) (200 Proof, ACS/USP grade, Ultrapure LLC). or the γ‐secretase inhibitor (GSI) DAPT (20 μM, Tocris Bioscience) for 1 h. An intramolecularly quenched fluorogenic peptide substrate (8 μM, Cat # 565764, Sigma‐Aldrich) was added, and tubes were incubated at 37°C overnight. Fluorescence was measured at 355 nm excitation/ 440 nm emission wavelength using SpectraMax 340PC spectrophotometer (Molecular Devices). Background fluorescence of the peptide probe was subtracted from all readings.

### 2.6 Cell transfection

The human Caveolin‐1 siRNA (cat# AM16708; Assay ID: 10392) and nonspecific negative control siRNA (scrambled control) (cat# AM4641) were purchased from Invitrogen; Thermo Fisher Scientific. The human caveolin‐1 pCDNA overexpression untagged plasmid was purchased from OriGene Biotechnology, Rockville, MD (cat# SC119082). Caveolin‐1 siRNA or pCDNA overexpression was performed according to the instructions of the Lipofectamine 3000 kit. Transfection efficiency was assessed using GFP‐tagged siRNA controls and was >65% in both HCAEC and HCASMC. After transfection for 48 h, the cells were treated with or without EtOH (25 mM), DLL4 (3 μg/ml), and DAPT (10 μM) for 24 h or as indicated. The knockdown and overexpression efficiency of caveolin‐1 transfection was confirmed by western blotting and/or RT‐PCR analysis.

### 2.7 Western blot

Total cell lysate was obtained using ice‐cold RIPA cell lysis buffer (cat# 89900, ThermoFisher Scientific) and 1X Protease and Phosphatase Inhibitor cocktail mixture. The extracted protein was quantified using Bio‐Rad protein assay kit II (cat# 5000002; Bio‐Rad). Proteins were separated by size using 4%–20% Mini‐Protean TGX Precast protein gels (cat# 4561094; Bio‐Rad) and then transferred to a nitrocellulose membrane (GE Healthcare Life Sciences). The protein‐blotted membrane was blocked with a 5% (w/v) skimmed milk solution in 0.1% (v/v) Tween‐20 for 1 h at room temperature in a rocking platform. Primary antibodies to caveolin‐1 (cat# 3238) and beta‐actin (cat# 4970) (Cell Signaling Technology) were incubated overnight at 4°C. The secondary antibody‐anti‐rabbit IgG, HRP conjugated (cat# 7074; Cell Signaling Technology) was incubated for 120 min at room temperature in rocking platform. Proteins were visualized with a SuperSignal west Pico Chemiluminescent Substrate (cat # 34580; ThermoFisher Scientific) and ChemiDoc XR Imaging System (Bio‐Rad).

### 2.8 Data analysis

Data are expressed as mean ± SEM. For all experiments, experimental points were performed at least in duplicate, with a minimum of three independent experiments performed on different days. An unpaired Student's *t*‐test was performed to determine differences between two groups, while multiple datasets were analyzed by ordinary one‐way ANOVA followed by a Tukey's or Dunnett's multiple comparison test (GraphPad Prism software V 9.1.0). A value of *p* ≤ 0.05 was considered significant.

## 3 RESULTS

### 3.1 Dose–response of EtOH on Notch signaling in human coronary artery ECs

**FIGURE 2:**
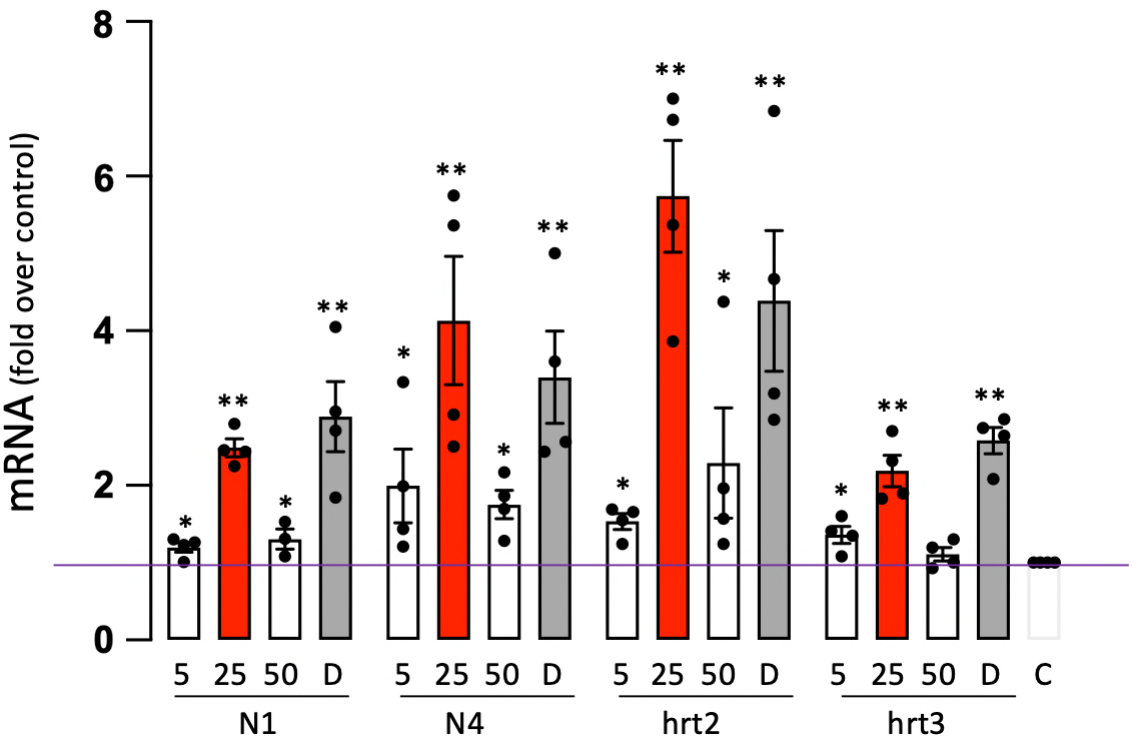
Ethanol (EtOH) stimulates Notch signaling in HCAEC. Notch receptors 1 and 4 (N1, N4) and target gene hrt2 and hrt3 mRNA levels determined by qRT‐PCR in HCAEC treated (24 h) without (control, C) or with EtOH (5 mM, 25 mM [red bars], or 50 mM) or Dll4, (D, 3 μg/ml). Data are mean ± SEM, *n* = 4, *p < 0.05, **p < 0.01 versus control.

Notch receptor and target gene expression were assessed by qRT‐PCR in human coronary artery endothelial cells (HCAEC) treated with or without different concentrations of EtOH (5, 25, and 50 mM to mimic levels achieved after light, moderate, and heavy drinking, respectively) or the Notch ligand delta‐like ligand 4 (Dll4, 3 μg/mL) as a control. EtOH treatment increased Notch receptor 1 and 4 (i.e., the receptor subtypes predominant in ECs) and Notch target gene hrt2 and hrt3 mRNA expression with the most robust response, similar to that of Dll4, elicited at 25 mM EtOH (Figure 2).

### 3.2 EtOH stimulates γ‐secretase activity in endothelial cells (HCAEC)

**FIGURE 3:**
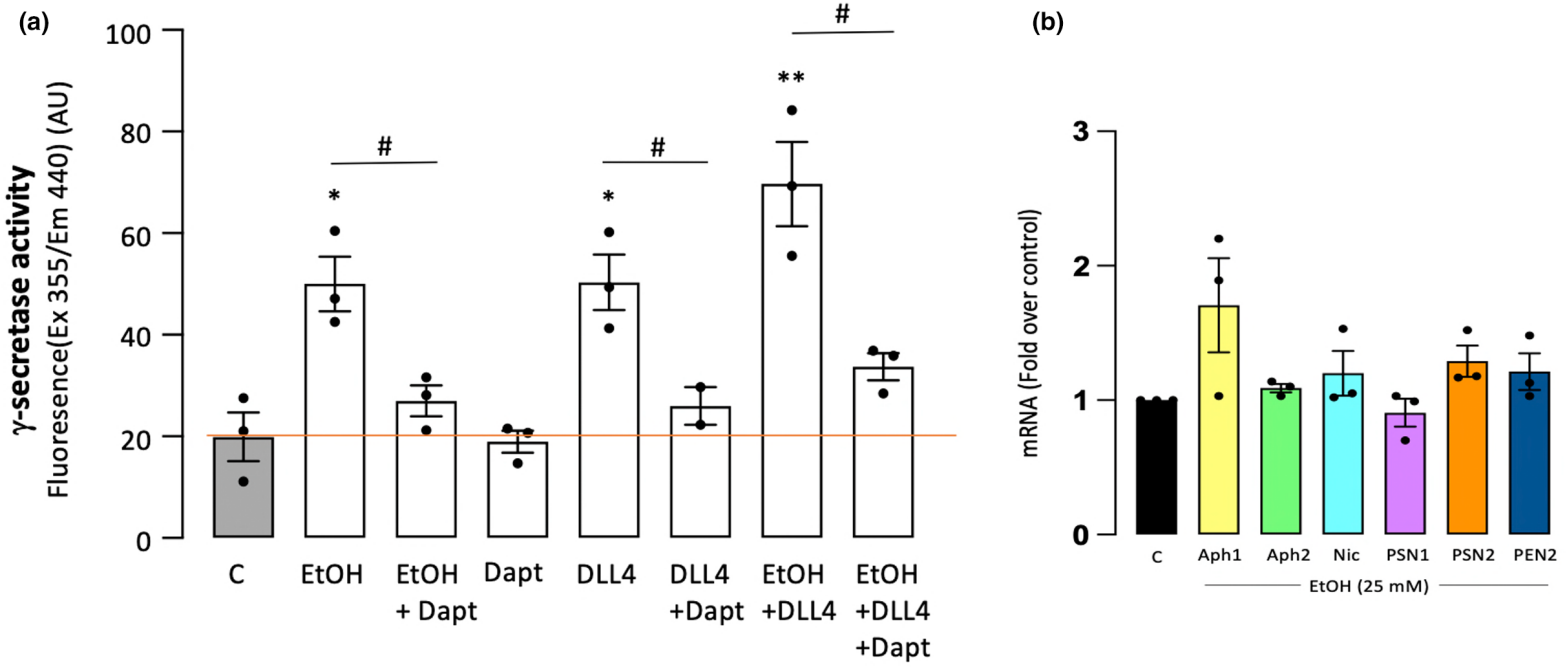
(a) Ethanol (EtOH, 25 mM) stimulates endothelial cell γ‐secretase activity. Gamma‐secretase activity in solubilized human coronary artery endothelial cell (HCAEC) membranes was measured using a fluorogenic peptide substrate (C‐terminal fragment of β‐APP) as described in Materials and Methods. DAPT was used as control γ‐secretase inhibitor (GSI). DLL4 = Notch ligand “delta like ligand 4.” (b) γ‐secretase subunit mRNA levels, determined by qRT‐PCR, in HCAEC treated (24 h) with or without EtOH (25 mM). Ethanol had no effect on mRNA levels of any of the subunits measured (i.e., Aph1, 2, Nicastrin (Nic), Presenilin 1, 2 (PSN), Pen2). C = control (no ethanol). Data are mean ± SEM, *n* = 3. *p < 0.05, **p < 0.01 versus C; #*p* < 0.05 +/− DAPT.

The effect of EtOH on γ‐secretase proteolytic activity in HCAEC‐solubilized membranes was determined as described in Methods. In this assay, increased fluorescence is indicative of γ‐secretase cleavage activity. Similar to the Notch ligand Dll4, treatment of HCAEC with EtOH (25 mM, 24 h) stimulated γ‐secretase activity >twofold above control levels, a response that was inhibited by the specific γ‐secretase inhibitor (GSI) DAPT (20 μM) (Figure 3a). The γ‐secretase multiplex is composed of subunits including presenilin (PSN), presenilin enhancer (PEN), nicastrin (NIC), and anterior pharynx defective (Aph). EtOH (25 mM, 24 h) had no effect on γ‐secretase subunit mRNA expression in HCAEC as determined by qRT‐PCR (Figure 3b).

### 3.3 EtOH inhibits caveolin‐1 expression in HCAEC

**FIGURE 4:**
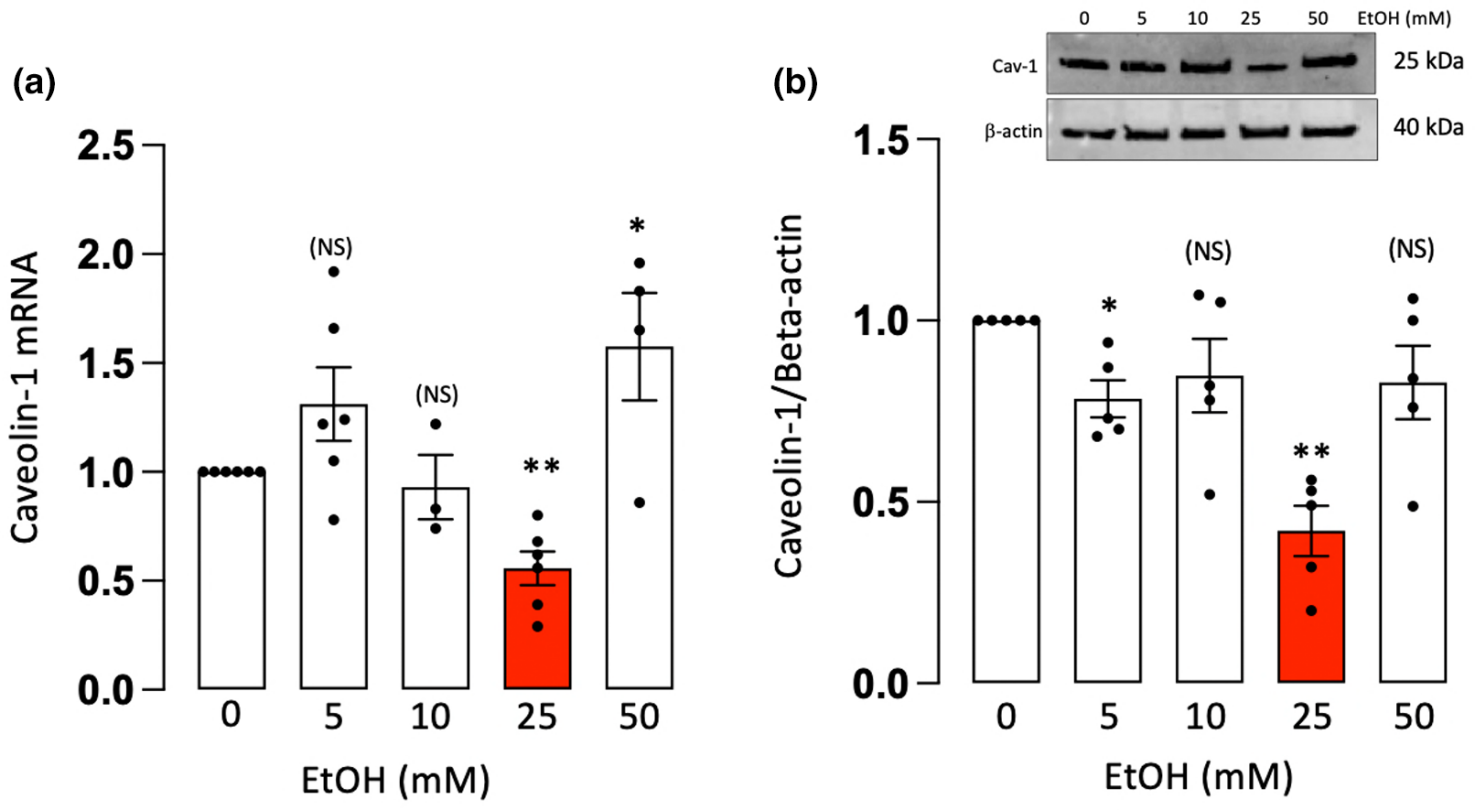
Ethanol inhibits caveolin‐1 expression in EC. (a) Caveolin‐1 mRNA levels, determined by qRT‐PCR, in HCAEC treated (24 h) with or without EtOH (5–50 mM). Data are mean ± SEM *n* = 3–6. (b) Caveolin‐1 protein levels determined by western blot. Cumulative data (mean ± SEM, *n* = 4–5) are shown together with a representative blot. β‐actin was used as a loading control. *p < 0.5, **p < 0.01, versus control (c). NS = not significant.

Caveolin‐1 can modulate cell signaling and has been implicated in the regulation of both Notch signaling and γ‐secretase activity in various cell types (Li et al., 2011; Wang et al., 2013; Zou et al., 2015). Therefore, caveolin‐1 (Cav‐1) mRNA and protein levels were determined, by qRT‐PCR and western blot, respectively, in HCAEC treated with or without EtOH (5–50 mM, 24 h). EtOH at 25 mM inhibited both Cav‐1 mRNA and protein levels in these cells, with little or no effect at the other concentrations tested (Figure 4).

### 3.4 EtOH inhibits caveolin‐1 expression in HCASMC

**FIGURE 5:**
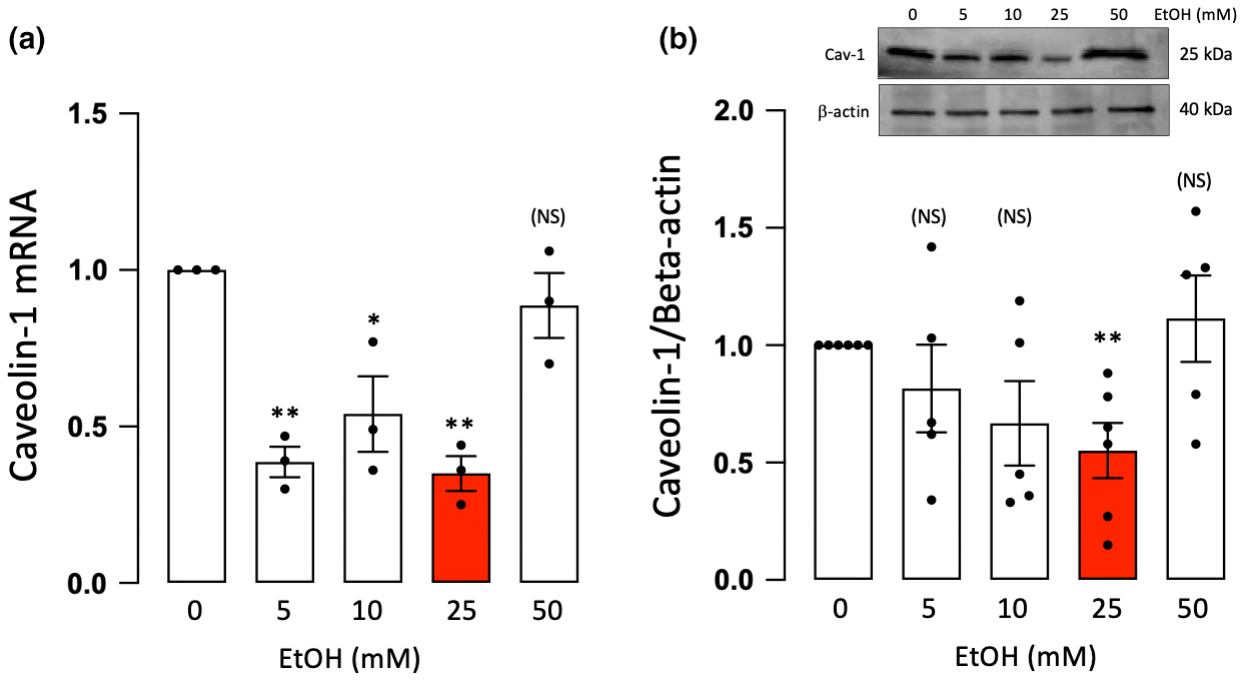
Ethanol inhibits caveolin‐1 expression in SMC. (a) Caveolin‐1 mRNA levels, determined by qRT‐PCR, in HCASMC treated (24 h) with or without EtOH (5–50 mM). Data are mean ± SEM *n* = 3–6. (b) Caveolin‐1 protein levels determined by western blot. Cumulative data (mean ± SEM, *n* = 4–5) are shown together with a representative blot. β‐actin was used as a loading control. *p < 0.5, **p < 0.01, versus control (c). NS = not significant.

Dose–response effects of EtOH on caveolin‐1 in arterial smooth muscle cells were also determined. Similar to the response in HCAEC, EtOH at 25 mM inhibited both Cav‐1 mRNA and protein levels in human coronary artery smooth muscle cells (HCASMC) (Figure 5). EtOH (5, 10 mM) also inhibited Cav‐1 mRNA levels in these cells.

### 3.5 EtOH's stimulation of γ‐secretase activity in EC is prevented by caveolin‐1 overexpression

**FIGURE 6:**
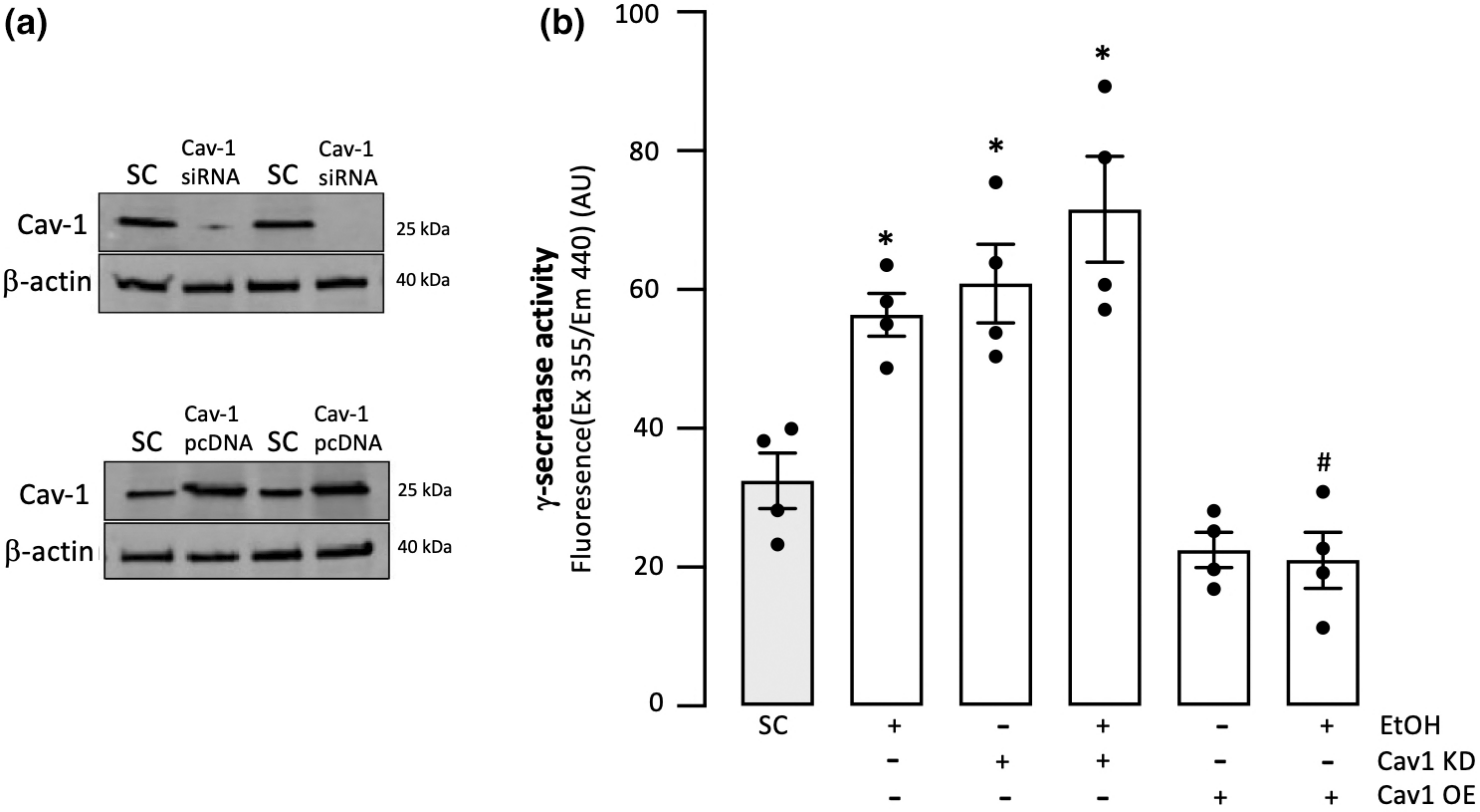
(a) Representative western blots showing reduced and increased caveolin‐1 (Cav‐1) protein in HCAEC following transfection with siRNA (top) or pcDNA (bottom), respectively. (b) Effect of Cav‐1 manipulation on γ‐secretase activity and on the EtOH γ‐secretase response in HCAEC. γ‐secretase activity was determined in solubilized membranes from HCAEC in which Cav‐1 was either knocked down (using Cav‐1 siRNA) or overexpressed (Cav‐1 pcDNA). Caveolin‐1 negatively regulates γ‐secretase activity in endothelial cells, and its overexpression blocks the stimulatory effect of EtOH on γ‐secretase in these cells. Data are mean ± SEM, *n* = 4. *p < 0.5 versus scrambled control (SC), #*p* < 0.01 versus EtOH alone.

The effect in arterial ECs of caveolin‐1 manipulation on γ‐secretase activity and on EtOH's effect was assessed in HCAEC in which caveolin‐1 had been either knocked down or overexpressed as described in Materials and Methods. Caveolin‐1 knockdown resulted in stimulation of γ‐secretase activity, whereas caveolin‐1 overexpression inhibited activity compared to controls (scrambled control) indicating that caveolin‐1 *negatively* regulates the enzyme in these cells (Figure 6). Furthermore, EtOH's stimulatory effect on γ‐secretase tended to be enhanced in HCAEC in which caveolin‐1 had been knocked down, whereas caveolin‐1 overexpression completely blocked it (Figure 6).

### 3.6 EtOH's inhibition of γ‐secretase activity in SMC is prevented by caveolin‐1 overexpression

**FIGURE 7:**
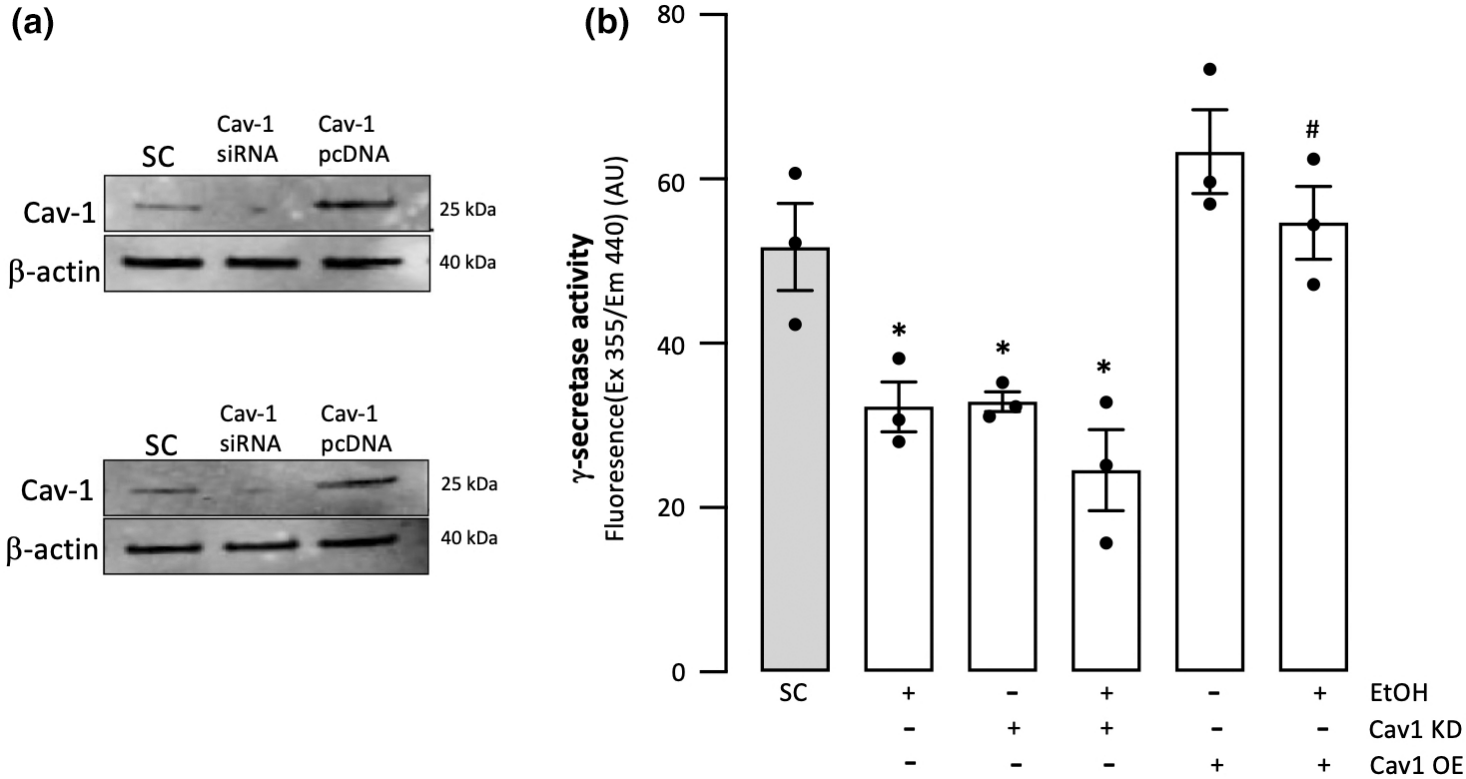
(a) Representative western blots showing reduced and increased caveolin‐1 (Cav‐1) protein in HCASMC following transfection with Cav‐1 siRNA and Cav‐1 pcDNA, respectively (two different treatments shown). (b) Effect of caveolin‐1 manipulation on γ‐secretase activity and on the EtOH γ‐secretase response in HCASMC. γ‐secretase activity was determined in solubilized membranes from HCASMC in which Cav‐1 was either knocked down (using Cav‐1 siRNA) or overexpressed (Cav‐1 pcDNA). Caveolin‐1 positively regulates γ‐secretase activity in smooth muscle cells, and its overexpression blocks the inhibitory effect of ethanol on γ‐secretase in these cells. Data are mean ± SEM, *n* = 3. *p < 0.5 versus scrambled control (SC), #*p* < 0.01 versus EtOH alone.

In HCASMC, caveolin‐1 knockdown significantly inhibited γ‐secretase activity, whereas caveolin‐1 overexpression tended to increase it, suggesting that caveolin‐1 *positively* regulates γ‐secretase activity in these cells (Figure 7). Furthermore, Cav‐1 overexpression prevented the inhibitory effect of EtOH on SMC γ‐secretase, whereas caveolin‐1 knockdown had no effect on it (Figure 7).

## 4 DISCUSSION

Here we report that alcohol (EtOH) at moderate levels stimulates γ‐secretase proteolytic activity in arterial ECs, an effect directly opposite to its effect on this protease in smooth muscle cells. Moreover, these differential effects of EtOH on γ‐secretase in the two vascular cell types are both mediated by caveolin‐1 inhibition.

γ‐secretase is a unique membrane‐embedded protease that cleaves the transmembrane domain of over 100 identified substrates, the most well‐appreciated of which are Notch and amyloid precursor protein (Guner & Lichtenthaler, 2020). In the case of Notch, the transmembrane domain cleavage of the Notch receptor by γ‐secretase is part of the cell signaling pathway and is necessary for signal transduction to occur. Specifically, cleavage of the receptor results in generation of a shorter secreted form, that is, the Notch intracellular domain (NICD), that can be routed to the nucleus (canonical pathway) to elicit transcription of target genes (Artavanis‐Tsakonas et al., 1999). Knockout of γ‐secretase is embryonic lethal and closely resembles the phenotypes seen with Notch1 receptor knockout suggesting that the role of γ‐secretase in Notch signaling may be one of its main biological functions (Wolfe, 2020).

Abundant evidence implicates dysregulation of Notch in the pathophysiology of cardiovascular disease (Aquila et al., 2019; Marracino et al., 2021; Redmond et al., 2013, 2014). Notch signaling modulates the functions of endothelial and smooth muscle cells, both of which are critical in dictating the balance between arterial health and pathology, being either protective or harmful depending on the cell context. For example, Notch 1 is mechanosensitive and is activated by laminar shear stress resulting in the upregulation of genes that preserve endothelial function (Briot et al., 2016; Mack et al., 2017). Moreover, we and others have shown that the protective effects of 17‐β‐estradiol and of alcohol against inflammatory‐induced endothelial dysfunction are, at least in part, Notch dependent (Fortini et al., 2017; Rajendran et al., 2021). With respect to smooth muscle cells, however, Notch activation is considered pro‐atherogenic as it promotes changes in SMC phenotype from differentiated (quiescent, contractile) to de‐differentiated (secretory, synthetic) resulting in cell growth and migration that facilitates intima‐medial thickening (IMT) and pathophysiological remodeling (Dave et al., 2022; Hatch et al., 2018; Havrda et al., 2006; Morrow et al., 2005; Sweeney et al., 2004).

Of interest, alcohol (EtOH) differentially regulates Notch signaling in EC and SMC, being stimulatory and inhibitory, respectively (Morrow et al., 2008, 2010), effects expected to be synergistically vascular protective. We previously showed that EtOH's inhibition of Notch in SMC occurred prior to NICD generation, and was in fact mediated by inhibition of γ‐secretase activity (Hatch et al., 2015). Here, we confirm that finding and additionally report for the first time that in contrast to its SMC effects, EtOH stimulates γ‐secretase activity and Notch signaling in EC with the most robust response achieved at “moderate level” 25 mM. While 50 mM EtOH also stimulated Notch signaling, its effect was much less than 25 mM EtOH, indicating a biphasic dose–response, suggestive of hormesis, that is, a different effect of low versus high doses. We have observed this frequently with EtOH and various vascular effects (Redmond unpublished observations). Low‐to‐moderate drinking can be considered 1–3 drinks per day (giving rise to BACs in the range 5–25 mM) where a standard drink in the USA contains 14 g of pure alcohol; this amount of alcohol (i.e., 14 g) is found in 12 ounces of beer, 5 ounces of wine, or 1.5 ounces of liquor. The National Institute of Alcohol Abuse and Alcoholism (NIAAA) currently defines “low risk drinking” for men as no more than four drinks on any single day and no more than 14 drinks per week, and for women as no more than three drinks on any single day and no more than seven drinks per week. A 25 mM blood alcohol level, the concentration of EtOH that caused the most robust stimulation of Notch and γ‐secretase activity in EC, is within the range that could be achieved by these “low risk drinking” daily limits. We note that in this study, cells were exposed to EtOH at the indicated concentrations for 24 h, with little‐to‐no metabolism occurring. This scenario does not mimic that in vivo following alcohol consumption where blood alcohol levels peak within 60 min, then start to fall. Thus, the translational relevance of our in vitro data to alcohol consumers is somewhat limited and requires further investigation.

Caveolae are discrete regions, observed by electron microscopy as small flask‐shaped invaginations, within the plasma membrane that regulate cholesterol transport and cell signaling processes including nitric oxide and calcium signaling, and play important roles in vascular homeostasis and disease (Li et al., 2005; Potje et al., 2020). Caveolin‐1, the major component of caveolae, is abundantly expressed in both EC and SMC. Its functional role is controversial in these two cell types (Chidlow Jr. & Sessa, 2010); it appears to have dual action in the vasculature impairing function in specific cases while also being essential to maintaining vascular homeostasis (Potje et al., 2020). Caveolin‐1 has been suggested as a possible therapeutic target for hypertension and atherosclerosis as its silencing prevents Angiotensin II (Ang II)‐induced pro‐atherogenic changes in both endothelial and smooth muscle cells, and Ang II‐induced pathological remodeling in mice (Forrester et al., 2017). Moreover, the relationship between caveolin‐1 and endothelial nitric oxide synthase (eNOS) is extremely important in vascular function. Specifically, loss of caveolin‐1 leads to persistent eNOS activation and increased nitric oxide (NO) in cells through loss of the caveolin‐1 inhibitory effect on eNOS (Chidlow Jr. & Sessa, 2010). NO is important in maintaining vascular homeostasis; it is a vasodilator and has antiproliferative, antioxidant and anti‐inflammatory properties (Cahill & Redmond, 2016).

**FIGURE 8:**
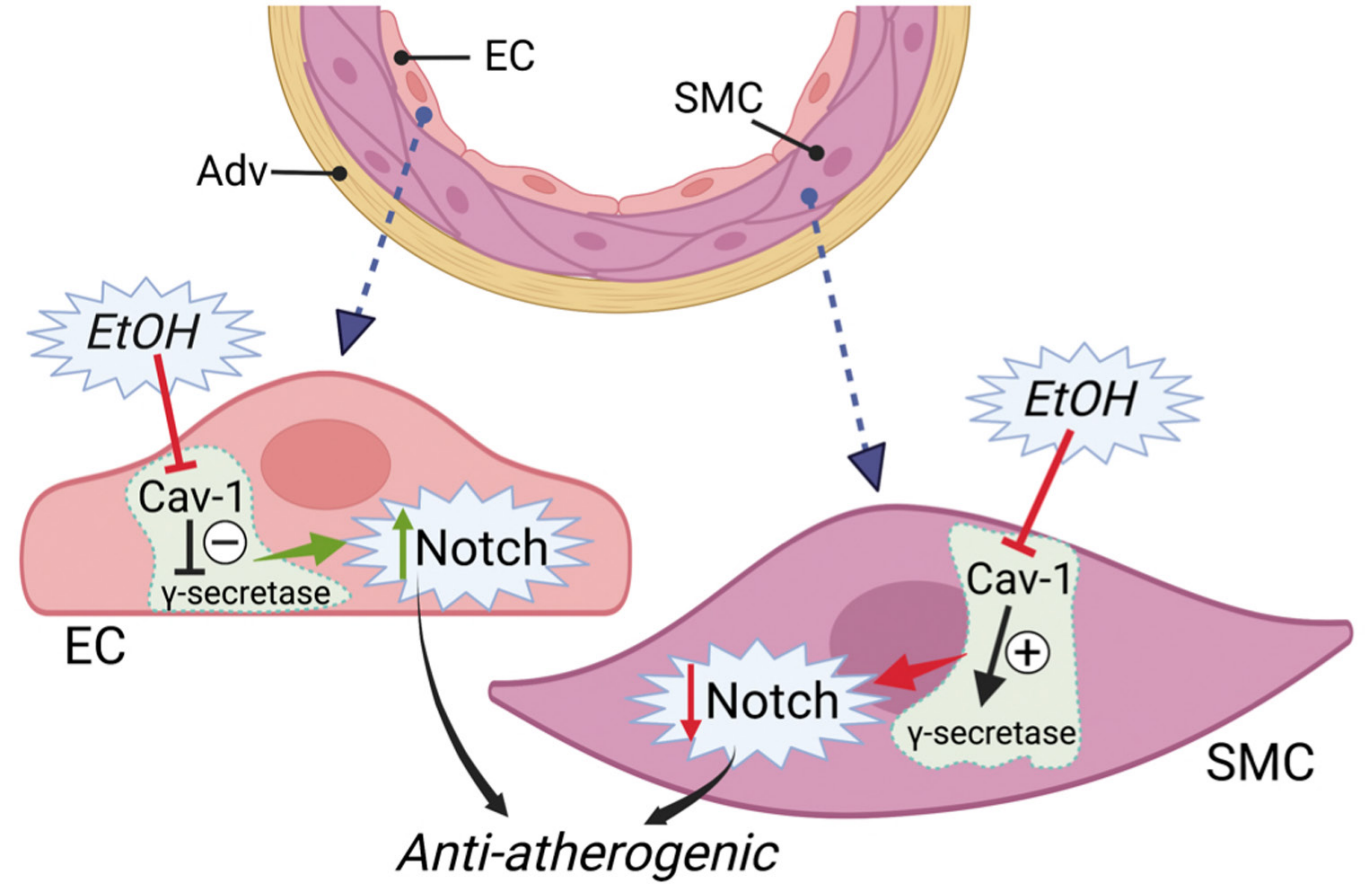
Alcohol (EtOH) inhibits caveolin‐1 (Cav‐1) expression in both endothelial cells (EC) and smooth muscle cells (SMC). Caveolin‐1 negatively regulates γ‐secretase activity in EC, whereas it positively regulates γ‐secretase activity in SMC. Thus, alcohol‘s inhibition of Cav‐1 expression in EC and SMC results in increased and decreased Notch signaling, respectively. These differential changes in Notch signaling in EC and SMC would be expected to act synergistically to maintain vessel homeostasis and prevent arteriosclerosis. Adventitia (Adv). Figure created using BioRender.

Our data demonstrate that EtOH at moderate levels inhibits caveolin‐1 mRNA expression and protein levels in both vascular cell types (EC, SMC) studied. The mechanism(s) mediating this alcohol effect was not determined. It may involve direct inhibition of gene transcription or posttranscriptional events by epigenetic processes (e.g., DNA methylation) or by noncoding RNAs, or indirect regulation of a pathway upstream of caveolin‐1 (Ciafre et al., 2019; Quest et al., 2008); further investigation is warranted in this regard. Caveolin‐1 inhibition by EtOH would be expected to increase eNOS activity and NO in EC (Chidlow Jr. & Sessa, 2010) and while not addressed in this study, we and others have previously reported such an effect (Hendrickson et al., 1999; Rocha et al., 2012). Interestingly, experimental manipulation of caveolin‐1 levels in arterial cells (i.e., by knockdown and overexpression) showed that while caveolin‐1 *negatively* regulates γ‐secretase activity in EC, it *positively* regulates it in SMC. Furthermore, EtOH's stimulatory effect on γ‐secretase activity was enhanced in EC in which caveolin‐1 had been knocked down, and completely blocked by caveolin‐1 overexpression. While in SMC, caveolin‐1 overexpression prevented the inhibitory effect of EtOH on SMC γ‐secretase, whereas caveolin‐1 knockdown had no effect on it. These data indicate that the differential effects of EtOH on γ‐secretase activity in EC and SMC are both mediated by caveolin‐1 inhibition which itself has opposite effects on γ‐secretase in the two vascular cell types (Figure 8). Taken in context with previous studies by us and others (Chidlow Jr. & Sessa, 2010; Fernandez‐Sola, 2015; Forrester et al., 2017; Li et al., 2009; Liu et al., 2011; Morrow et al., 2008, 2010; Rajendran et al., 2021), the inhibitory effect of EtOH on caveolin‐1 expression and the subsequent opposing effects of that on γ‐secretase/Notch signaling in arterial endothelial and smooth muscle cells may underlie, in part, the protective effects of moderate drinking on atherosclerosis. Future studies to confirm a key role for caveolin‐1 in mediating the effect of moderate alcohol consumption on arterial disease in vivo are warranted. Moreover, given the numerous substrates of γ‐secretase identified to date including amyloid precursor protein, investigation of the concept that alcohol may also affect other diseases such as Alzheimers disease and cancers via this mechanism is also warranted.

## AUTHOR CONTRIBUTIONS

NKR, PAC, and EMR conceived and designed the research. NKR and WL performed experiments. NKR and EMR analyzed data and prepared figures. NKR, PAC, and EMR interpreted results of experiments. NKR, PAC, and EMR drafted, edited, and revised the manuscript. NKR, WL, PAC, and EMR approved final version of manuscript.

## CONFLICT OF INTEREST

The authors declare that there is no conflict of interest.
